# Connectogram-COH: A Coherence-Based Time-Graph Representation for EEG-Based Alzheimer’s Disease Detection

**DOI:** 10.3390/diagnostics15111441

**Published:** 2025-06-05

**Authors:** Ehssan Aljanabi, İlker Türker

**Affiliations:** Department of Computer Engineering, Karabuk University, Karabuk 78050, Turkey; salman.ehssan@gmail.com

**Keywords:** functional brain networks, graph representations, EEG classification, Alzheimer’s disease detection (ADD), graph mining, time series classification

## Abstract

**Background:** Alzheimer’s disease (AD) is a neurological disorder that affects the brain in the elderly, resulting in memory loss, mental deterioration, and loss of the ability to think and act, while being a cause of death, with its rates increasing dramatically. A popular method to detect AD is electroencephalography (EEG) signal analysis thanks to its ability to reflect neural activity, which helps to identify abnormalities associated with the disorder. Originating from its multivariate nature, EEG signals are generally handled as multidimensional time series, and the related methodology is employed. **Methods**: This study proposes a new transformation strategy that generates a graph representation with time resolution, which handles EEG recordings as relatively small time windows and converts these segments into a similarity graph based on signal coherence between available channels. The retrieved adjacency matrices are further flattened to form a 1-pixel image column, which represents the coherence activity from the available electrodes within the given time window. These pixel columns are concatenated horizontally for all available sliding time windows with 50% overlap, resulting in a grayscale image representation that can be input to well-known deep learning architectures specialized for images. We name this representation Connectogram-COH, a coherence-based version of the previously proposed time graph representation, Connectogram. **Results**: The experimental results demonstrate that the proposed Connectogram-COH representation effectively captures the coherence dynamics of multichannel EEG data and achieves high accuracy in detecting Alzheimer’s disease. The time graph images serve as robust input for deep learning classifiers, outperforming traditional EEG representations in terms of classification performance. **Conclusions**: Connectogram-COH offers a powerful and interpretable approach for transforming EEG signals into image representations that are well suited for deep learning. The method not only improves the detection of AD but also shows promise for broader applications in EEG-based and general time series classification tasks.

## 1. Introduction

Alzheimer’s disease (AD) is a chronic neurodegenerative disorder that disrupts memory, cognition, and behavior and is the leading cause of dementia in older adults. It presents as progressive loss of neural function, eventually impairing a person’s ability to perform daily tasks [1,2]. AD has become one of the leading causes of death globally [3], and the socioeconomic burden of the disease continues to rise. The World Alzheimer Report projects the number of people living with AD to reach 75 million by 2030 and a staggering 131 million by 2050 [4], underscoring the urgency of developing scalable, reliable, and non-invasive diagnostic tools.

Currently, diagnosis often relies on a combination of clinical evaluations, neuropsychological tests such as the Mini-Mental State Examination (MMSE) and Montreal Cognitive Assessment (MoCA), and structural or molecular imaging [5]. However, these assessments are typically initiated after symptom onset and are limited by subjective judgment, variability in symptom presentation, and dependence on human expertise. The invasive nature and high cost of imaging methods like PET and MRI further limit their accessibility, particularly for population-wide screening in low-resource settings

Given these constraints, researchers are shifting their attention to non-invasive, inexpensive, and scalable alternatives, notably electroencephalography (EEG). EEG is uniquely suited for early diagnosis due to its superior temporal resolution, ease of use, and sensitivity to subtle neural dynamics [6,7,8]. Importantly, EEG allows for repeated measurements over time, making it ideal for tracking disease progression and therapeutic response. Conventional EEG analysis often involves decomposing signals into canonical frequency bands—delta (0.5–4 Hz), theta (4–8 Hz), alpha (8–13 Hz), beta (13–25 Hz), and gamma (25–45 Hz)—each associated with distinct cognitive and physiological functions [9,10]. Abnormalities in these bands, particularly alpha and theta rhythms, are widely reported in AD patients. Transform methods like fast Fourier transform (FFT) [11], Welch’s power spectral density (PSD) [12], discrete wavelet transform (DWT) [13], and empirical mode decomposition (EMD) [14] have been extensively used to analyze these frequency components and extract informative features for classification.

More recently, a paradigm shift toward functional connectivity (FC) and network science has emerged [15,16]. These approaches focus not only on local activity but also on inter-regional communication within the brain. Metrics such as phase-locking value (PLV), coherence, and amplitude envelope correlation are used to quantify synchrony between EEG channels [17,18]. The resulting connectivity matrices represent the brain as a complex graph, where nodes are electrode sites (or brain regions) and edges represent statistical dependencies or coupling strength. Notably, studies show that AD patients often exhibit disrupted global efficiency and reduced clustering in their functional brain networks [19,20], reflecting a breakdown in integrative cognitive processes.

This graph-based conceptualization has promoted the application of graph theory and network neuroscience in AD research. Measures such as global efficiency, betweenness centrality, and clustering coefficients have shown significant differences between healthy and AD-affected individuals [17,21,22]. For instance, Kabbara et al. demonstrated that alpha band connectivity disruptions in resting-state EEGs were positively correlated with cognitive impairment levels in AD, indicating potential use as a biomarker.

These findings have aligned with advances in deep learning (DL), which allow models to automatically learn patterns from high-dimensional data without manual feature engineering. Early work, such as that by Alves et al. (2021), utilized deep neural networks (DNNs) on EEG-derived connectivity matrices to classify AD and schizophrenia with impressive accuracy, outperforming traditional EEG-based classifiers [23]. Building on this, Demir et al. (2021) introduced graph neural networks (GNNs), which respect the spatial relationships between electrodes and incorporate structural information into learning, allowing models to exploit topological features of the brain network—a significant advancement over CNNs, which assume regular grid-like inputs [24].

Beyond static graph representations, the need to capture temporal evolution in brain connectivity has gained traction. Dynamic functional connectivity—where network properties change over time—can reveal transient synchronization events and early biomarkers missed by static analyses. Studies like that by Gupta et al. (2022) have employed tensor decomposition to model EEG data across time, space, and frequency dimensions, recovering latent temporal patterns that align with disease stages [25]. Similarly, the LEAD model proposed by Wang et al. (2025) introduced a scalable pretraining strategy using contrastive learning across large EEG datasets, achieving state-of-the-art performance in subject-independent AD classification [26].

Another noteworthy development is the move toward interpretable AI. Ajra et al. (2023) emphasized the use of shallow CNNs and interpretable connectivity metrics like amplitude envelope correlation (AEC) to classify EEG signals in AD and frontotemporal dementia (FTD), achieving over 94% accuracy [27]. Their work highlights the diagnostic potential of frequency-specific network features. Similarly, Fruehwirt et al. (2018) employed Bayesian neural networks to provide not only predictions but also uncertainty estimates, which are critical for clinical decision making [28].

Despite these successes, challenges remain, especially regarding signal artifacts, inter-subject variability, and generalizability. Recent approaches attempt to address these via subject-independent validation, data augmentation, or by focusing on subcortical source-localized signals [29]. Some models, like the one proposed by Sunkara et al. (2024), even explore alternative neural architectures (e.g., Kolmogorov–Arnold networks) to enhance classification robustness under noisy conditions [30].

The current study builds upon this body of work by proposing a novel methodology for representing time-evolving EEG graphs as 2D images. This transformation preserves the temporal dynamics of functional connectivity while enabling the application of powerful CNN architectures originally developed for image classification. While previous methods primarily utilized static graphs or flattened features [9,11,12,13,14,17,31,32], our approach allows for the concatenation of dynamic connectivity matrices across sliding time windows. This results in a “graph sequence image” that captures both spatial and temporal fluctuations in brain synchronization.

Such methodology aligns with trends in both multimodal EEG representation and efficient deep learning, enabling reduced model complexity without compromising classification performance. It also provides a framework that is generalizable to other time series domains, suggesting broad applicability beyond AD diagnosis. The integration of neuroscience-informed signal processing and interpretable machine learning underscores the potential of our approach to bridge the gap between research and clinical utility.

## 2. Data and Methodology

### 2.1. Dataset

The proposed methodology was evaluated in an openly available EEG dataset from the OpenNeuro data repository [33], comprising recordings from 88 participants. EEGs were recorded using 19 scalp electrodes placed according to the international 10–20 system (Fp1, Fp2, F7, F3, Fz, F4, F8, T3, C3, Cz, C4, T4, T5, P3, Pz, P4, T6, O1, and O2) and two mastoid reference electrodes (A1 and A2). All recordings followed a standardized clinical protocol, with participants seated comfortably and instructed to keep their eyes closed. The data were sampled at 500 Hz with a resolution of 10 μV/mm.

The dataset included three groups: 36 individuals with Alzheimer’s disease (AD; 20 female), 23 individuals with frontotemporal dementia (FTD; 9 female), and 29 cognitively normal (CN) participants (18 female). The average ages were 66.4 years (SD = 7.9) for the AD group, 63.6 years (SD = 8.2) for the FTD group, and 67.9 years (SD = 5.4) for the CN group. Cognitive functioning was assessed using the Mini-Mental State Examination (MMSE), with mean scores of 17.75 (SD = 4.5) for AD, 22.17 (SD = 8.22) for FTD, and 30 for CN, indicating moderate impairment in the dementia groups and normal cognition in controls. The AD group had a median disease duration of 25 months (IQR: 24–28.5).

Each EEG recording lasted approximately 13.5 min for AD participants (range: 5.1–21.3 min), 12 min for the FTD group (range: 7.9–16.9 min), and 13.8 min for the CN group (range: 12.5–16.5 min) [33,34]. These demographic and clinical details help to contextualize the stage of disease progression and support the interpretation of EEG patterns identified through machine learning analysis.

### 2.2. Data Processing for Time Graph Conversion

To enable classification using deep learning, the raw EEG recordings were first transformed into time graph image representations, referred to as Connectogram-COH. This transformation involved segmenting multichannel EEG signals into fixed-length, non-overlapping segments and subsequently converting each into a series of functional brain connectivity graphs.

First of all, the data consisting of multichannel EEG recordings from 19 electrodes were subdivided into fixed length segments (10, 20, or 30 s) without any overlap to generate a pool of independent EEG signals available for the classification task. This process provided the augmentation of the EEG dataset from 88 recordings into 6938, 3446, or 2285 segments, respectively, where each segment was labeled the same as its original full-length recording and had the same multivariate nature. These independent segments were the raw sources to be converted into time graph (grayscale image) representations.

We strictly ensured that all data segments derived from a given subject were assigned exclusively to either the training or the testing set within each fold. To enforce this, we adopted a subject-wise k-fold cross-validation strategy, where the data were split at the subject level, not at the segment level. The segmentations were performed after subject-level splitting, ensuring complete isolation between subjects in the training and testing sets. This meant that no segments from the same subject appeared in both the training and testing sets in any cross-validation fold. Applying zero overlap between segments also secured independence between segments and avoided data leakage. All of these steps collectively ensured the validity of our evaluation protocol and the reliability of the reported classification performance.

Next, each fixed-length segment was handled as a separate and independent data recording. To capture the fine-grained temporal dynamics of brain activity, a sliding window approach was applied. A window length of 0.4 s and overlap of 0.2 s (50% overlap) were employed to subdivide the data into time windows. This resulted in a sequence of short-duration multichannel EEG signals within each segment, ready for graph conversion.

Then, each 0.4 s window was transformed into a functional connectivity graph by computing coherence values between every pair of EEG channels. Coherence is a frequency domain measure that quantifies the degree of synchronization between two signals, providing insight into functional interactions between brain regions. The coherence metric represents the level of mutual information or synchronization of neural activity across the nodes, while the nodes correspond to physical brain areas. The mathematical background of the coherence metric between equal-length signals x(t) and y(t) can be defined using the cross-spectral density function P_xy_ and the power spectral density functions P_xx_ and P_yy_, as given in Equation (1) [18].

(1) $$Coh=\frac{{\left|P_{xy}\right|}^{2}}{P_{xx}P_{yy}}$$

**Figure 1:**
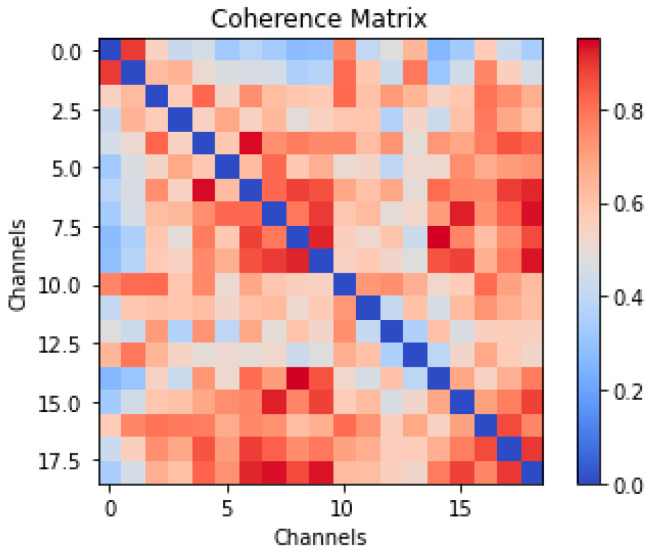
The visualization of an adjacency matrix that represents the functional brain network, derived from a 0.4 s time window of a 19-channel EEG recording.

This process produces a symmetric 19 × 19 coherence matrix called the adjacency matrix for each window, representing the functional network of brain regions. Figure 1 presents a 19 × 19 coherence matrix, which visualizes the functional connectivity for a 0.4 s EEG window.

In the adjacency matrix, each element represents the coherence between a pair of electrodes defining a connection weight between EEG channels (signals from independent electrodes), as visualized in Figure 1. In this functional brain graph, the nodes represent distinct brain regions or areas, often defined by anatomical or functional parcellation (e.g., regions from the cerebral cortex, subcortical structures), while the edges represent functional connections between these nodes, reflecting statistical relationships or interactions. Such graphs offer insight into the dynamic interactions among brain areas during different cognitive or physiological states.

The achieved adjacency matrix has a symmetric nature caused by the equal coherence values achieved in two directions for a signal pair. Therefore, the upper triangle of this matrix forms a meaningful representation for the corresponding time window. This upper triangle is later flattened to achieve a vector of size 171 × 1, which represents the connectivity pattern derived from a single time window.

**Figure 2:**
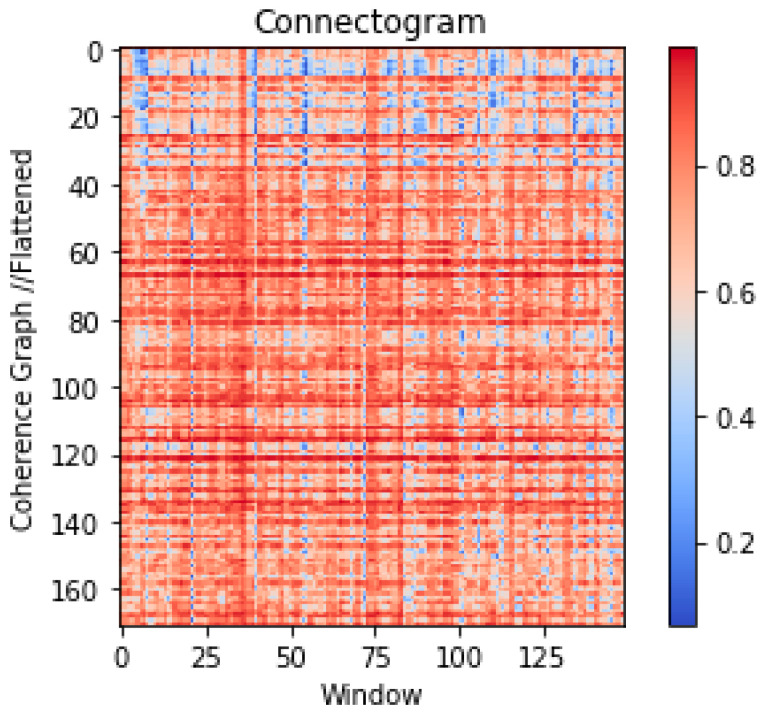
The Connectogram-COH image derived from a 30 s multichannel recording. Each pixel column represents a flattened graph representation of the 0.4 s time window, forming the vertical dimension of the image. These 1-pixel flattened vectors, tiled horizontally for each time window, form the time resolution of the image on the horizontal axis.

For each segment (10, 20, or 30 s), these flattened representation vectors are tiled horizontally, which results in 149 pixels of horizontal resolution. Therefore, the resulting time graph representation shown in Figure 2 has a resolution of 171 × 149 pixels for a 30 s segment. We named this representation Connectogram-COH, as a coherence-based variety of the recently proposed Connectogram for time series [35].

**Figure 3:**
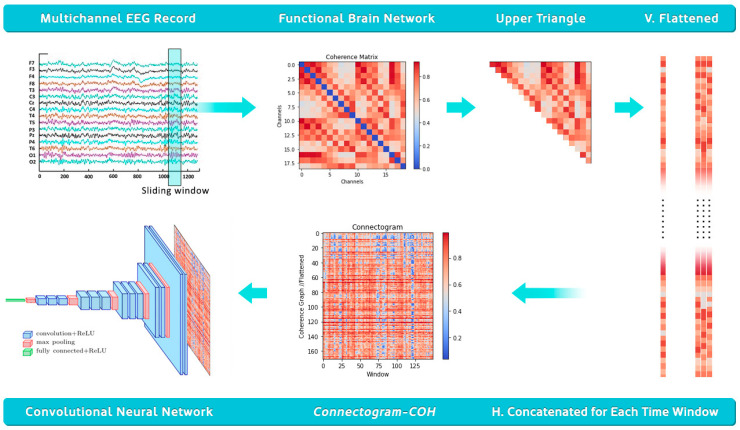
Illustration of the entire methodology presented. The time graph conversion is applied to equal-length EEG recordings, specifically 10, 20, or 30 s segments in this study. These recordings are handled in sliding time windows with a length of 0.4 s and 50% overlap (0.2 s). For each time window, a graph representation is generated based on the signal coherence values between EEG channels, corresponding to the edge weights, where each EEG channel (electrode) is a single node. Since the adjacency matrix of this graph is symmetrical, we extract the upper triangle and flatten it to obtain a vertical vector. For each sliding time window, a vertical graph representation is achieved, serving as a 1-pixel column that will be concatenated horizontally to generate a time graph representation. The resulting image, named Connectogram-COH, can be input to a well-known convolutional neural network model to perform the classification task.

These image-like representations are finally passed to suitable deep learning architectures such as CNN or transfer learning models, leading to successful classification results, as presented in the coming sections. The entire methodology is illustrated in Figure 3.

### 2.3. Experimental Setup

The experimental setup consisted of trials for various lengths of segmentation of the original recordings as 10, 20, or 30 s. For each segment, we provided the same class label as the original recording, and we generated a separate Connectogram-COH image for each of these data segments. Having applied this set of various segmentation lengths, we achieved 6938, 3446, and 2285 Connectogram-COH images and their corresponding class values. The segment length did not affect the dimension of the adjacency matrix, since it was determined by the number of EEG channels, which was 19 for the dataset used. The vertical dimension of the resulting Connectogram-COH remained the same, accordingly.

**Figure 4:**
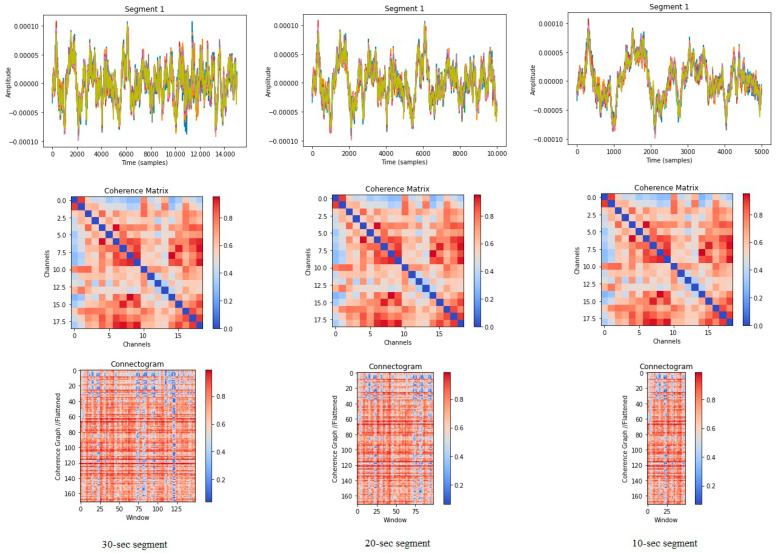
The Connectogram-COH for “30”, “20”, and “10” s segments.

However, the horizontal dimension scaled linearly with the size of the segment length, which determined the number of available sliding fixed-length windows. As a result, Connectogram-COH had the resolution of 171 × 149, 171 × 99, and 171 × 49 pixels for the 30, 20, and 10 s segments, respectively, as illustrated in Figure 4.

We tested the classification performance of the Connectogram-COH images across a variety of deep learning architectures, such as CNN, ResNet, VVG16, InceptionV3, EfficientNetB7, and DenseNet121. We also performed experiments for a range of batch sizes (4 to 512) and epochs (20 to 100) for the best performer learning model, as explained in the next section. The learning models used are briefly described below.

#### 2.3.1. Convolutional Neural Network (CNN)

A convolutional neural network (CNN) is a deep learning architecture specifically designed to process structured data, particularly images and visual inputs. It emulates the human brain’s visual processing through convolutional layers that automatically identify features such as edges, textures, and patterns. These layers utilize filters (kernels) that move across the input to generate feature maps, emphasizing key characteristics. Pooling layers allows to downsample the spatial dimensions, enhancing computational efficiency and resilience to minor input variations. Finally, fully connected layers integrate the extracted features to perform predictions. CNNs are extensively applied in image classification, object detection, and segmentation tasks due to their capability to learn hierarchical data representations. The custom CNN architecture employed in this study is detailed in Table 1.

#### 2.3.2. Residual Network (ResNet)

ResNet is a deep learning model designed to overcome the issue of vanishing gradients in very deep networks. It incorporates the concept of ‘residual learning’ by utilizing skip connections, which allow the model to bypass intermediate layers and transmit the input directly to deeper layers. This technique enables the network to learn the residual, or the difference, between the input and the output, rather than attempting to directly learn the output. As a result, ResNet prevents performance degradation in deeper networks, allowing the training of extremely deep models with hundreds or even thousands of layers. ResNet has proven highly effective in image recognition tasks and forms the basis for numerous advanced architectures [36]. There exists a variety of ResNet models, such as ResNet-50, ResNet-18, etc., but our model does not match any of these exact versions. It shares elements with smaller ResNet models (like ResNet-18/34) in terms of basic residual blocks, which makes it a “shallow ResNet” model, as described in Table 2.

#### 2.3.3. VGG-16

VGG-16 is a convolutional neural network (CNN) architecture that is mostly preferred for its simplicity and effectiveness in image recognition tasks. It comprises 16 layers with trainable parameters, including 13 convolutional layers and 3 fully connected layers. The network employs small 3 × 3 filters in all convolutional layers, stacked to deepen the network and capture intricate features. Pooling layers are interspersed to reduce spatial dimensions, while the fully connected layers at the end are responsible for classification. Despite having a relatively large number of parameters, the straightforward design of VGG-16 has established it as a benchmark model in computer vision, widely applied in transfer learning and feature extraction [37].

#### 2.3.4. Inception v3

Inception v3 is an advanced deep convolutional neural network designed for efficient and accurate image classification. It integrates innovative architectural features from the Inception family, optimizing performance while reducing computational cost. It leverages Inception modules, which stack filters of different sizes (e.g., 1 × 1, 3 × 3, and 5 × 5) to capture multi-scale features, all while maintaining low computational costs. The model introduces techniques like factorized convolutions (splitting larger convolutions into smaller ones, e.g., 3 × 3 into two 1 × 3 and 3 × 1), label smoothing to prevent overfitting, and an auxiliary classifier for gradient flow improvement. With 42 layers and approximately 23 million parameters, Inception v3 provides a balance between depth and efficiency, delivering state-of-the-art performance on tasks such as ImageNet classification [37].

#### 2.3.5. EfficientNet-B7

EfficientNet-B7, the largest model in the EfficientNet family, optimizes performance and computational efficiency through a compound scaling approach. This method systematically scales the network’s depth, width, and resolution, allowing the model to achieve state-of-the-art accuracy while requiring fewer parameters than traditional architectures. It incorporates advanced techniques such as squeeze-and-excitation blocks to improve feature extraction, swish activation functions for smoother gradients, and depthwise separable convolutions to reduce computational costs. With approximately 66 million parameters, EfficientNet-B7 achieves remarkable accuracy in image classification tasks, such as ImageNet, while maintaining a balance between performance and computational cost [37].

#### 2.3.6. DenseNet-121

DenseNet-121 is a convolutional neural network that introduces dense connectivity between layers to improve feature propagation and reduce redundancy. Unlike traditional architectures, where each layer connects only to the next, DenseNet establishes direct connections between every layer in a feed-forward manner. This dense connectivity enhances gradient flow, optimizes parameter usage, and mitigates the vanishing gradient problem. DenseNet-121 features 121 layers, organized into dense blocks and transition layers, with each layer receiving inputs from all preceding layers. This structure reduces the number of parameters while maintaining high accuracy. DenseNet-121 is particularly efficient for image classification tasks, achieving improved performance on benchmarks like ImageNet with fewer parameters compared to other models [37].

## 3. Results and Discussion

We first tested the classification performance of the generated time graph representations across the mentioned deep learning models to determine the best performing model. These experiments were conducted on the available three classes: control (CN), Alzheimer’s disease (AD), and frontotemporal dementia (FTD). For each experimental setup, a set of 5 experiments was conducted to achieve reliable results, which are presented in Table 3.

However, some of the experiments could not be executed since the aspect ratio of the images did not match the requirements of the learning models, being far from a square-like output. The experiments implied that the ResNet model outperformed the rest of the models, with 99.49% mean accuracy, while the custom CNN model scored the second-best accuracy (98.63%) after ResNet. Other models failed to compete with these models. The second major outcome of these experiments was that better accuracy results were achieved for lower-length data segments, which resulted in smaller connectograms on the horizontal scale.

**Figure 5:**
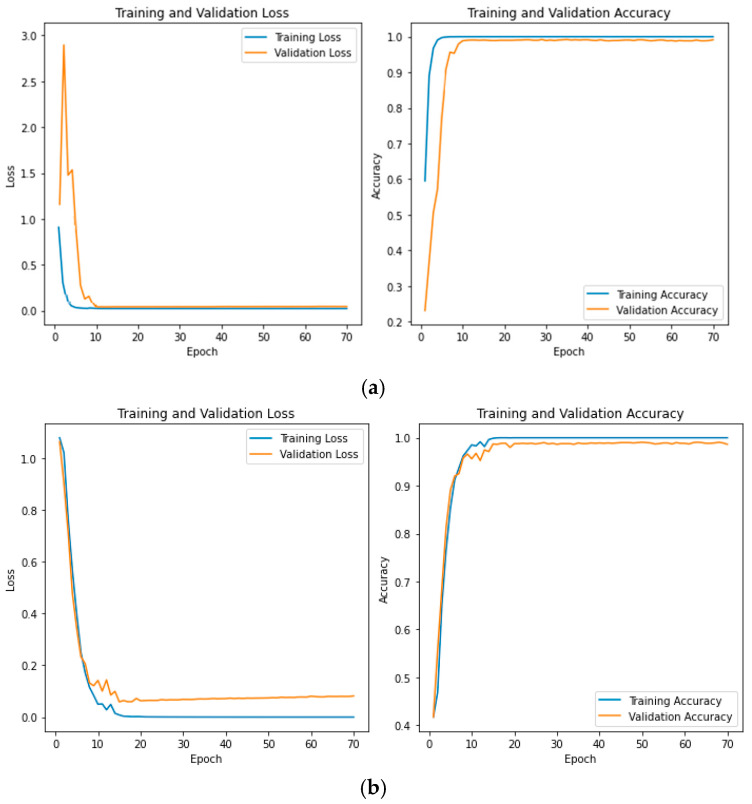
Learning curves for (**a**) CNN and (**b**) ResNet classifiers.

For the two-class case, ResNet achieved 99.53% accuracy for AD vs. CN, 99.50% for FTD vs. CN, and 99.45% for AD vs. FTD. Finally, the 5-fold mean accuracy for ResNet in the three-class scenario was 99.33%, with a standard deviation of 0.25. The learning curves for CNN and ResNet shown in Figure 5 demonstrate good generalization capability, with close alignment between training and validation accuracy, indicating no evidence of overfitting.

**Figure 6:**
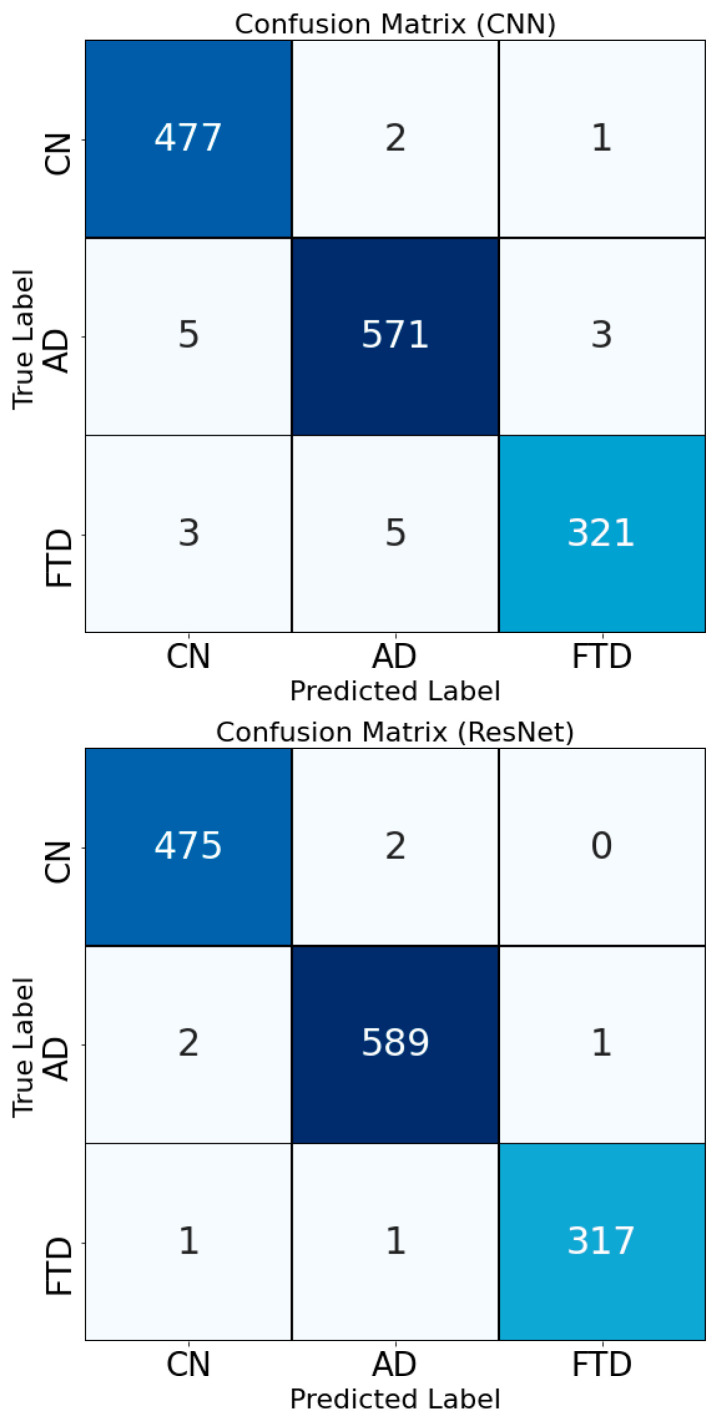
Confusion matrices for CNN and ResNet classifiers.

Confusion matrices for the first two models, CNN and ResNet, are also presented in Figure 6. The two models showed similar patterns of classification accuracy while their differences originated from mislabeled instances by CNN in the CN and AD classes. Very few misclassified instances in ResNet side demonstrated that the model had a very strong and robust performance for Connectogram-COH images.

**Figure 7:**
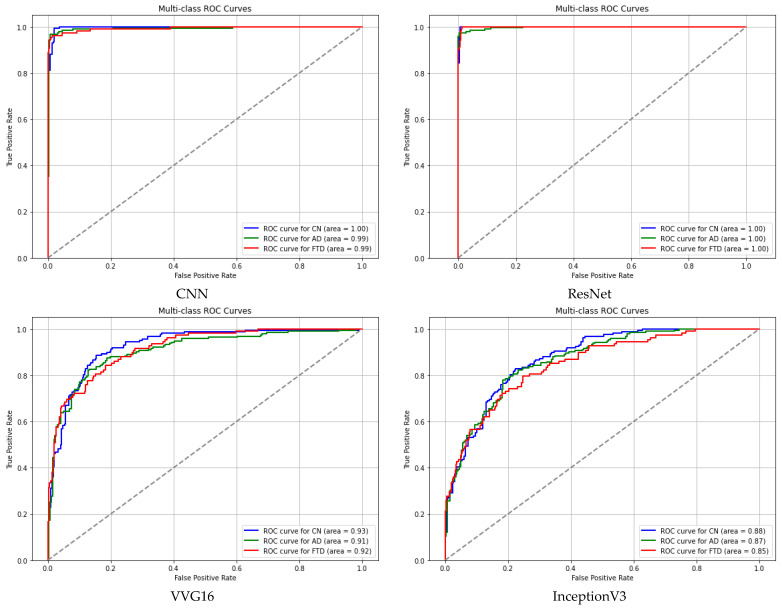

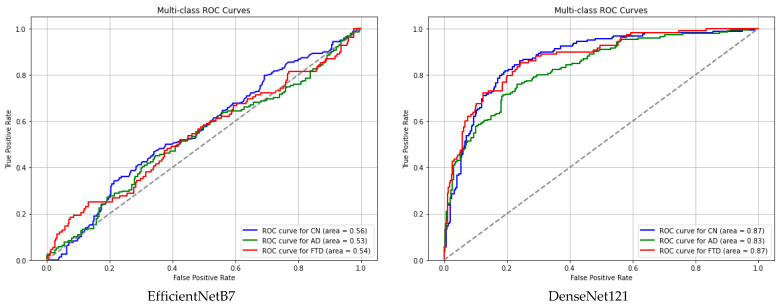
ROC curves of each of the algorithms used in this study.

The classification reports for the models tested are shown in Table 4, while Table 5 and Figure 7 present the ROC curves and the areas under the ROC curves for each algorithm employed.

Having determined the best performing model as ResNet, we fixed the architecture as ResNet and tried to fine-tune the framework. We repeated each experimental setup by subdividing the original data into different equal-length segments as 10, 20, and 30 s without any overlap, in compliance with the strategy commonly used for detecting brain disorders using EEG signals [38,39]. This segmentation strategy, beyond providing equal-size data inputs to the learning models, has the advantage of extending the available number of samples for a given dataset, which in turn meets the demand for high data volume by a deep learning model. The dataset employed in this study originally included 485.5 min of AD, 276.5 min of FTD, and 402 min of CN recordings which are extended into a total number of 6938, 3446 and 2285 equal-size 410 bins for 10, 20 and 30-s segment lengths. As a result, a sufficient number of independent samples was achieved to feed the deep neural network model. The zero-overlap segmentation procedure also enhanced the integrity and reliability of both percentage split and k-fold validation due by securing non-overlapping training and test sets.

Table 6 presents the average accuracy achieved for a variety of parametric values, ranging from 4 to 512 for batch size and 20 to 100 for epoch size, after 5 trials for each setup were performed.

The experiments yielded promising results for different batch sizes from one set to another, generally appearing for small-to-moderate batch sizes in the range 4 to 64. This was because choosing a small batch size such as (4, 8, 16, 32) may lead to fuzzy estimates of gradients, as the weights are updated based on a small number of examples each time. But it may be useful in avoiding overfitting and it contributes to speeding up in some cases. It helps to improve the generalizability of the model. On the other hand, choosing a large batch size such as (64, 128, 256, 512) may provide more stable and accurate estimates of gradients because a larger number of examples is used at each step. It can also reduce the “noise” in the gradients and speed up convergence, but it requires more memory and computational power. Sometimes, it may lead to poor generalizability if the size is not set appropriately. The same goes for the number of epochs; using too few epochs means training for too short a time and the model may not learn the data well enough, leading to poor performance (this is called underfitting). Conversely, if we specify too many epochs, the model may start to overfit the training data, meaning it will overlearn small details in the data, leading to poor performance on the test data.

Another important factor to note is the change in the number of images from one segment length to another. This was clearly shown when using a length of 30 s, where the number of images was 2285 and the resolution of each image was 171 × 149 pixels. By contrast, when using a length of 10 s, the number of images was up to 6638 with a relatively low image resolution of 171 × 49 pixels. Therefore, the quality of the test differed slightly. Finally, we can recommend the use of small-to-moderate batch size values and epochs, since the generated time graph representations had the ability to serve as robust input over a wide range of parameters; however, limiting the segment length values to 10 or 20 s would give better accuracy regardless of the other parameters tested. The robustness of the methodology also makes it suitable for detecting a wide range of brain disorders, such as autism, Parkinson’s, epilepsy, etc.

Unlike most studies performed recently that handled binary classification tasks such as AD-FTD, AD-CN, or FTD-CN, our study handles a three-class problem, which critically affected the classification metrics. The results presented, which include the classification of frontotemporal dementia in addition to Alzheimer’s disease, make the approach valuable and promising for several multiclass classification tasks, such as cardiac disorders that range from 4 to 20 or more classes, in case multi-lead recordings are available.

Recent research on neurodegenerative disease detection using electroencephalography (EEG) signals has introduced a variety of innovative approaches tailored for specific classification tasks. Dogan et al. [40] proposed a binary classification model for Alzheimer’s disease (AD) detection, employing a graph-based methodology inspired by the primate brain connectome to extract discriminative features from EEG signals. Miltiadous et al. [10,41] extended this work by addressing the differentiation between AD and frontotemporal dementia (FTD), leveraging robust classification techniques and novel neural architectures, such as DICE-Net, which combines convolutional and transformer-based models. Araujo et al. [42] introduced a smart data-driven system for AD detection, demonstrating the utility of advanced feature extraction in EEG-based studies, while Gomez et al. [43] focused on multiclass classification tasks involving AD, mild cognitive impairment (MCI), and healthy controls, highlighting the potential of spontaneous EEG activity for accurate classification. Safi et al. [11] emphasized the importance of early detection, utilizing Hjorth parameters to effectively distinguish AD from control groups. Khatun et al. [44] developed a single-channel EEG approach based on speech-evoked brain responses for detecting mild cognitive impairment, showing the feasibility of simplified setups. Beyond EEG, Gordon et al. [45] explored clinical and MRI data to measure disease progression in frontotemporal lobar degeneration.

Despite these advancements, most prior studies are limited to binary classification tasks or smaller datasets, with minimal focus on visualizing connectivity patterns over time. Our study bridges these gaps by introducing a time graph representation methodology for a three-class classification task, distinguishing AD, FTD, and control groups. This approach generates a unique output image where the horizontal axis represents time and the vertical axis reflects dynamic connectivity patterns across brain regions, providing an innovative visualization of neural activity. Furthermore, our work leverages a significantly larger EEG dataset than many existing studies, enabling better generalization and robustness. By combining time graph imaging with advanced classification techniques, our study offers a novel perspective on understanding and differentiating neurodegenerative diseases, paving the way for more comprehensive and scalable methodologies in EEG-based diagnostics.

In comparison to prior studies employing three-class classification approaches, including those by Lopes et al. [46], Gomez et al. [43], Ieracitano et al. [47], and Xia et al. [48], which achieved competitive results in differentiating Alzheimer’s disease (AD), mild cognitive impairment (MCI), and healthy controls, our proposed strategy demonstrates superior performance in classification accuracy and robustness. Lopes et al. [46] combined convolutional neural networks (CNNs) with saliency maps and EEG modulation spectra to enhance interpretability and diagnostic accuracy for AD. Gomez et al. [43] utilized spontaneous EEG activity for multiclass classification, emphasizing the efficacy of automated feature extraction methods. Similarly, Ieracitano et al. [47] developed a CNN-based model that employed two-dimensional spectral representations of EEG recordings to effectively classify dementia stages. Xia et al. [48] introduced a deep pyramid CNN architecture tailored for EEG signals, showcasing its potential in AD detection with high precision. While these studies have made significant contributions, they primarily focus on either specific architectures or unique representations of EEG data.

Both the CNN and residual network (ResNet) architectures integrated into our framework offer significant advantages in handling complex EEG patterns, which are converted into grayscale image representations. However, a key limitation lies in the reliance on a fixed number of electrodes, which constrains the vertical dimension of the resulting image. A central design principle of the proposed methodology is the creation of square-like images to enable compatibility with certain deep learning architectures. This limitation necessitates careful balancing of parameters that affect the vertical and horizontal dimensions of the image—particularly the selection of appropriate segmentation lengths and windowing strategies—to ensure comparable dimensions across both axes. However, there lies a key strength of the methodology if the pixel aspect ratio constraint is loosened, introducing an inherent flexibility in adapting to different EEG setups. Various window-length values, resulting in an imbalanced width/height aspect ratio, would also represent the original signal activity with various temporal characteristics embedded into the graph representation. With the aid of concurrent advancements in the AI domain, these output images with imbalanced aspect ratios can also be processed efficiently by deep learning models. For example, when working with 20, 32, or 64 channels (c), the resulting coherence (or adjacency) matrices scale accordingly to 20 × 20, 32 × 32, and 64 × 64. These matrices are then flattened into vectors of sizes 190 × 1, 496 × 1, and 2016 × 1, respectively (can be calculated as c(c − 1)/2 to reflect the size of the upper triangle of the adjacency matrix), effectively capturing the connectivity patterns within each time window regardless of the montage. This same adaptable framework applies to changes in segmentation and windowing parameters. By adjusting these inputs, the method maintains compatibility with various EEG acquisition setups, enabling generalization across datasets with different channel counts and recording configurations. Overall, while the current implementation is optimized for a specific configuration, the underlying principles can be extended through scaling strategies, dynamic preprocessing, and architectural adjustments. This flexibility enhances the broader applicability of our approach in both research and clinical EEG contexts.

Another potential concern is that flattening the upper triangle of the coherence matrix may discard essential topological properties of spatiotemporal graphs. To address this, we employed a sliding window approach, segmenting the data into discrete time windows and computing a coherence matrix for each segment. This strategy yields a time series of matrices rather than a single static representation, thereby enabling the capture of dynamic fluctuations in connectivity patterns over time. To reduce redundancy while retaining key information, we extracted only the upper triangular portion of each coherence matrix. This conservative linear representation preserves the exact pairwise connectivity values and their ordering—features that are often the most informative for classification tasks in clinical applications. Although this approach does not explicitly preserve certain abstract topological properties (e.g., spatial node configurations), it maintains the core connectivity structure necessary for effective downstream analysis.

The reason for not implementing and testing the Connectogram-COH images on graph-based learning models is that these models expect pure graph structures (adjacency matrices) as input, while our model outputs flattened graphs concatenated on the horizontal axis, resulting in time graph representations. These images rather resemble the structure of power spectrogram images that are well known in the literature, which are commonly input to CNN-like architectures for classification tasks.

Importantly, we enforced strict subject-wise cross-validation to eliminate the risk of data leakage, which could otherwise lead to artificially inflated classification performance. Specifically, all data segments from a given subject were assigned exclusively to either the training or the testing set within each fold. We adopted a subject-wise k-fold cross-validation strategy, where data splitting was performed at the subject level, followed by segmentation. This ensured that no data from the same subject appeared in both the training and testing sets in any fold. Additionally, to further mitigate the risk of data leakage, all preprocessing and segmentation procedures were conducted independently within each fold. We also avoided using overlapping segments, which helps to reduce the likelihood of high similarity between samples in the training and testing sets. These precautions ensured the validity and generalizability of our model evaluation.

It is important to note that we recognize that recent literature increasingly explores transformer models, graph neural networks, and multimodal data fusion. Spatiotemporal GNNs (ST-GNNs), in particular, provide a powerful framework for modeling dynamic and spatial interactions by leveraging the complete graph structure over time, often resulting in superior performance in both predictive and interpretive tasks. However, these models typically require significant design effort, including the construction of the graph itself and the manual definition of node features.

By contrast, our study aims to demonstrate that a well-structured convolutional neural network (CNN)-based architecture—specifically ResNet—can achieve competitive, and in some cases superior, performance when combined with biologically meaningful input features, such as coherence-based connectograms. With appropriate preprocessing and rigorous validation, this approach proves highly effective, as evidenced by its performance compared to more complex transformer-based models such as that in [10], despite using the same dataset. Our contribution lies in showing that architectural simplicity, when paired with strong signal representation, can rival or even surpass more elaborate pipelines under certain conditions. Moreover, our method integrates seamlessly with traditional high-performance classifiers such as XGBoost. Notably, despite its streamlined structure, our approach achieved an excellent classification accuracy of 99.49%.

Table 7 presents a list of recent studies that have addressed the same problem together with the details of the datasets and methodology in comparison with the current study to provide insights about the improvements provided. It is important to note that the performance comparisons should be interpreted in the context of varying study designs, dataset sizes, and classification tasks. The given results indicate that our methodology can outperform all of the recent studies handling the same task. Meanwhile, some referenced works use much smaller datasets (fewer subjects, such as in [35,44,49,50]), which can lead to overfitting or inflated performance. The success of the methodology is more apparent among the studies handling relatively large datasets, including more than a hundred subjects, and multiclass classifications. We can conclude that the repeatability of success for all pairs of classes is mostly caused by the high representative capability of the generated time graph representations.

## 4. Model Deployment

As shown in Table 8, this compact deep learning model features just under 1 million trainable parameters, achieving a balanced trade-off between capability and efficiency. Its lightweight architecture enables deployment across a wide range of platforms—including desktops, mobile devices, and embedded edge hardware—without the need for substantial computational resources.

With a small memory footprint, the model is especially well suited for storage-constrained environments such as mobile applications, embedded systems, and specialized domains like medical, industrial IoT, and mobile computing. The minimal storage requirements also facilitate cloud-based deployment, allowing for fast downloads and seamless updates via APIs.

In terms of performance, the model demonstrates high throughput, making it ideal for batch inference tasks such as offline data processing, bulk analytics, and backend pipelines where real-time responsiveness is not critical. The inference latency supports near real-time processing, which suffices for many interactive applications. However, further optimization may be required for ultra-low-latency scenarios, such as brain–computer interfaces (BCIs) or real-time biomedical signal analysis, where milliseconds can be mission-critical.

## 5. Limitations and Future Directions

While our methodology has demonstrated effectiveness, several limitations merit attention:

- Fixed Electrode Count: The current design supports only 19-channel EEG. For high-density arrays, the image size may become unwieldy. Future work could use graph pooling, dimensionality reduction, or adaptive montages to scale effectively.
- Aspect Ratio Issues: Some CNN models (e.g., InceptionV3, EfficientNetB7) struggle with the elongated shape of connectograms. Padding, resizing, or using models that accept non-square inputs can improve compatibility.
- Explainability: Explainable AI (XAI) techniques applied to Connectogram-COH images to highlight which regions most influence the model’s predictions would give valuable insights to the study. Additionally, for EEG coherence graphs, graph-specific explainability approaches could offer insight into which connections or brain regions are most critical to the classification. These additions would help clinicians to better understand and trust the model’s decisions, and we view them as a vital direction for future work.

The need for more flexible data representations would encourage future work that would:

- Implement dynamic window sizing and normalization to better handle varying EEG lengths, sampling rates, and channel counts.
- Benchmark the method on multiple public datasets, using fine-tuning or domain adaptation as needed.
- Explore graph-based models (such as GNN) to retain spatial and temporal structures for improved performance.
- Apply transfer learning to adapt pretrained models to smaller, clinical datasets.

In the long term, we aim to translate this work into real-world clinical applications by partnering with clinicians to conduct prospective studies, compare model predictions with expert diagnoses, and ensure alignment between model outputs and established diagnostic criteria. These steps will help to bridge the gap between research and practice, fostering trust and utility in AI-assisted neurological diagnostics.

## 6. Conclusions

The current study proposes a novel time graph representation technique that can be applied to any multidimensional time series data. The graph conversion methodology relies on assuming each channel of EEG data as a node, while the signal coherence values between each signal pair represent the weight between corresponding nodes. In contrast to recent approaches converting EEG recordings into a static graph, the proposed transformation technology also captures the time dependency of the functional connectivity graph, resulting in a time graph representation that can be handled as a grayscale image and input to image-related deep learning architectures. The methodology is tested on a task related to the detection of Alzheimer’s disease by employing a variety of deep learning architectures. The promising results indicate the potential of the transformation strategy to be employed in any type of multidimensional time series data by extending the static graph representation approach into a time-dependent variant by enhancing a suitable graph conversion strategy that is coherent to the signal’s nature. The proposed framework was applied to a dataset available in the OpenNeuro data repository [19], labelled as Alsheimer’s disease (AD), frontotemporal dementia (FTD), or control (CN) groups, and overperformed current state-of-art methodologies in the field at a significant level. As a result, this study upgrades the capability of accurate classification of multidimensional time series data, specifically focusing on the diagnosis of Alzheimer’s disease but has similar potential for detecting several disorders, such as autism, Parkinson’s, epilepsy, etc. We also believe that introducing the time dimension to brain connectivity graphs not only captures the connectivity patterns of signal activity between brain regions but also represents the time-dependent temporal activity, providing a trustworthy basis for future research and clinical diagnosis.

## Figures and Tables

**Table 1:** The custom CNN model used in this study.

Layer	Type	Output Shape	Details
Input	Input Layer	(None, 171, 149, 1)	-
Conv2D	Convolutional Layer	(None, 169, 147, 32)	Filters: 32, Kernel: 3, Stride: 1
MaxPooling2D	Pooling Layer	(None, 84, 73, 32)	Pool Size: 2
Conv2D	Convolutional Layer	(None, 82, 71, 64)	Filters: 64, Kernel: 3, Stride: 1
MaxPooling2D	Pooling Layer	(None, 41, 35, 64)	Pool Size: 2
Conv2D	Convolutional Layer	(None, 39, 33, 128)	Filters: 128, Kernel: 3, Stride: 1
MaxPooling2D	Pooling Layer	(None, 19, 16, 128)	Pool Size: 2
Flatten	Flatten Layer	(None, 38,912)	-
Dense	Fully Connected Layer	(None, 128)	Units: 128
Dense	Fully Connected Layer	(None, 3)	Units: 3 (Output classes)

**Table 2:** The custom ResNet model used in this study.

Layer	Type	Output Shape	Details
Input	Input Layer	(None, 171, 149)	-
Conv1D	Convolutional Layer	(None, 86, 64)	Filters: 64, Kernel: 3, Stride: 2
BatchNorm	Batch Normalization	(None, 86, 64)	-
Activation	ReLU Activation	(None, 86, 64)	-
MaxPooling1D	Pooling Layer	(None, 43, 64)	Pool Size: 2
Residual Block 1	2x Conv + Add	(None, 43, 64)	Skip connection, Filters: 64, Kernel: 3
Residual Block 2	2x Conv + Add	(None, 22, 128)	Strided conv for downsampling, Filters: 128
Residual Block 3	2x Conv + Add	(None, 11, 256)	Strided conv for downsampling, Filters: 256
GlobalAvgPooling	Global Avg Pooling	(None, 256)	-
Dense	Fully Connected Layer	(None, 3)	Units: 3 (Output classes)

**Table 3:** Mean accuracy metrics for the learning models tested, performed for segmentations into 10, 20, and 30 s scenarios. For each scenario, the duration of the sliding window (0.4 s) and overlap (50%) remained the same. Some of the experiments could not be handled and are noted as N/A because the aspect ratio of the images was not suitable for the corresponding learning models.

Model/Segment Len.	30 s	20 s	10 s
CNN	96.28	97.82	98.63
ResNet	98.59	99.09	99.49
VVG 16	77.68	73.04	73.55
InceptionV3	71.33	N/A	N/A
EfficientNetB7	41.79	41.73	41.71
DenseNet121	69.36	69.42	N/A

**Table 4:** Mean classification report for the learning models tested, performed for segmentations into 30-s scenario. For each scenario, the duration of the sliding windows (0.4 s) and overlap (50%) remain the same.

		Accuracy	Precision	Recall	F1-Score	Support
ResNet	CN	0.9859	0.9875	0.9875	0.9875	158
	AD	0.9859	0.9843	0.9741	0.9792	191
	FTD	0.9859	0.9623	0.9808	0.9714	108
CNN	CN	0.9672	0.9625	0.9747	0.9686	158
	AD	0.9672	0.9737	0.9686	0.9711	191
	FTD	0.9672	0.9626	0.9537	0.9581	108
VVG16	CN	0.7877	0.7268	0.8924	0.8011	158
	AD	0.7877	0.8471	0.7539	0.7978	191
	FTD	0.7877	0.8065	0.6944	0.7463	108
InceptionV3	CN	0.7155	0.7143	0.7278	0.7210	158
	AD	0.7155	0.7404	0.8063	0.7719	191
	FTD	0.7155	0.6591	0.5370	0.5918	108
EfficientNetB7	CN	0.4179	0.0000	0.0000	0.0000	158
	AD	0.4179	0.4179	1.0000	0.5895	191
	FTD	0.4179	0.0000	0.0000	0.0000	108
DenseNet121	CN	0.6980	0.7066	0.7468	0.7262	158
	AD	0.6980	0.7198	0.6859	0.7024	191
	FTD	0.6980	0.6481	0.6481	0.6481	108

**Table 5:** The ROC curves for the learning models tested, performed for segmentations into 30 s scenario. For each scenario, the duration of the sliding window (0.4 s) and overlap (50%) remained the same.

	CN	AD	FTD
ResNet	0.9995	0.9989	0.9999
CNN	0.9963	0.9924	0.9934
VVG16	0.9253	0.9066	0.9160
InceptionV3	0.8757	0.8658	0.8489
EfficientNetB7	0.5593	0.5288	0.5429
DenseNet121	0.8716	0.8320	0.8726

**Table 6:** Mean accuracy metrics of the ResNet model with our approach using different segment lengths, epochs, and batch sizes, with each cell representing the average output of 5 executions.

	Epochs\Batch_Size	4	8	16	32	64	128	256	512
10 s segments	20	98.77	99.49	98.99	98.41	97.62	97.47	94.52	71.54
	50	99.63	99.42	99.06	99.49	99.49	98.41	95.24	88.61
	70	99.35	99.27	99.27	99.49	99.56	99.42	96.58	89.21
	100	99.42	99.27	99.20	99.49	99.63	98.99	97.81	91.18
20 s segments	20	93.18	95.94	97.82	98.63	99.27	89.27	71.15	50.28
	50	99.42	99.42	98.40	98.82	98.84	97.24	89.19	70.43
	70	99.13	99.27	98.98	99.27	99.42	98.81	89.27	79.56
	100	98.26	99.56	99.42	98.99	97.39	95.79	89.56	77.82
30 s segments	20	96.49	98.90	98.03	98.03	93.93	81.16	62.45	58.29
	50	97.37	97.59	98.24	97.81	98.03	95.18	89.27	70.32
	70	98.03	97.59	98.03	98.24	98.24	96.14	87.23	71.33
	100	98.90	98.86	98.41	98.41	98.59	94.21	88.55	71.99

**Table 7:** Comparison of the current study with recent studies. (AD: Alzheimer’s disease, MCI: mild cognitive impairment, CN: healthy control, FTD: frontotemporal dementia, CV: cross-validation.

Author(s)	Year	Classifier	Size of the Dataset	No. of Channels	Segment Length (s)	Folds for CV	Accuracy
Gomez et al. [43]	2018	MLP	111	19	-	-	AD-MCI-CN: 78.43
Xiaojun & Haibo [49]	2019	CNN	12	64	-	1–10	95.04
Ieracitano et al. [47]	2019	CNN	189	19	5	8	AD-CN: 92.95 AD-MCI: 84.61 MCI-CN: 91.88 AD-MCI-CN: 83.33
Khatun et al. [44]	2019	ERP SVM	23	1	-	-	87.9
Ismail et al. [51]	2019	CNN	60	10	16	-	AD-CN: 92.52 MCI-CN: 90.36
Wen et al. [52]	2020	CNN	39	19	-	5	92.92
Siluy et al. [50]	2020	ELM SVM KNN	27	19	2	10	ELM: 98.78 SVM: 97.41 KNN: 98.19
Cassani et al. [53]	2020	SVM	54	20	8	-	78.7
Safi & Safi [11]	2021	SVM KNN RLDA	86	20	8	-	SVM: 95.79 KNN: 97.64 RLDA: 97.02
Miltiadous et al. [41]	2021	Meny	28	19	5	10	AD-CN: 78.58 FTD-CN: 86.30
Huggins et al. [54]	2021	CNN	141	20	5	10	99.3 AD 98.3 MCI 98.8 CN
Amini et al. [55]	2021	CNN	192	19	-	-	82.3
Dogan et al. [40]	2022	KNN	23	16	-	10	92.1
Araujo et al. [42]	2022	SVM	38	19	5	-	AD-CN: 81 MCI-CN: 79
Ding et al. [4]	2022	Meny	301	60	15	5	AD-CN: 72.43 AD-MCI: 69.11 MCI-CN: 59.91
Xia et al. [48]	2023	CNN	100	19	-	5	AD-MCI-CN: 97.10
Wu et al. [56]	2023	STAE	53	16	1	-	96.30
Lopes et al. [46]	2023	CNN SVM	54	20	8	-	87.3
Miltiadous et al. [10]	2023	DICE-net	88	19	30	5	AD-CN: 83.28 FTD-CN: 74.96
Zhou et al. [57]	2024	STCGRU	27	19	5	10	MCI: 99.95
Parra et al. [58]	2024	CNN	668	32	-	5	CN-ADA: 97.49 CN-ADM: 97.03
Our study	2024	ResNet	88	19ch	30 20 10	5	AD-CN: 99.53 AD-FTD: 99.45 FTD-CN: 99.50 AD-FTD-CN: 99.41

**Table 8:** An overview of performance outputs.

Metric	Value	Comment
Trainable Parameters	979,715	Medium-sized model. Can run on desktop, mid-level mobile, or embedded edge devices.
Model Size	11.45 MB	Compact enough for mobile apps or cloud API deployment.
Inference Latency	147.4 ms (1 sample)	Good for near real-time processing, but may need optimization for ultra low-latency apps (e.g., BCI, live EEG).
Throughput	234.4 samples/s	Very efficient batch processing—good for offline or background inference.

## Data Availability

The original data presented in the study are openly available in OpenNeuro.org at https://doi.org/10.18112/openneuro.ds004504.v1.0.1.
